# Macrophages: Regulators of the Inflammatory Microenvironment during Mammary Gland Development and Breast Cancer

**DOI:** 10.1155/2016/4549676

**Published:** 2016-01-17

**Authors:** Nicholas J. Brady, Pavlina Chuntova, Kathryn L. Schwertfeger

**Affiliations:** ^1^Microbiology, Immunology and Cancer Biology Graduate Program, University of Minnesota, Minneapolis, MN 55455, USA; ^2^Department of Lab Medicine and Pathology, University of Minnesota, Minneapolis, MN 55455, USA; ^3^Masonic Cancer Center, University of Minnesota, Minneapolis, MN 55455, USA

## Abstract

Macrophages are critical mediators of inflammation and important regulators of developmental processes. As a key phagocytic cell type, macrophages evolved as part of the innate immune system to engulf and process cell debris and pathogens. Macrophages produce factors that act directly on their microenvironment and also bridge innate immune responses to the adaptive immune system. Resident macrophages are important for acting as sensors for tissue damage and maintaining tissue homeostasis. It is now well-established that macrophages are an integral component of the breast tumor microenvironment, where they contribute to tumor growth and progression, likely through many of the mechanisms that are utilized during normal wound healing responses. Because macrophages contribute to normal mammary gland development and breast cancer growth and progression, this review will discuss both resident mammary gland macrophages and tumor-associated macrophages with an emphasis on describing how macrophages interact with their surrounding environment during normal development and in the context of cancer.

## 1. Introduction to Macrophages

As a cell of the innate immune system, macrophages play critical roles in both host defense against pathogens and proper tissue development. During embryonic development, a population of macrophages derived from yolk sac hematopoiesis can be found throughout the organism and are thought to contribute to the populations of tissue-resident macrophages in the adult. This process occurs prior to the induction of hematopoiesis in the bone marrow, strongly suggesting a unique origin and function for these embryonic macrophages [1, 2]. Additionally, embryonically derived, tissue-resident macrophages have been found in a diverse array of organs and tissues, including the mammary gland, and the maintenance of these populations does not require monocyte precursors [3]. Postnatally, however, the multistep differentiation program that leads to mature macrophages begins in the bone marrow with hematopoietic stem cells (HSCs) [4]. These c-kit^+^/Sca-1^+^/lineage (Lin)^−^ HSCs give rise to two distinct multipotent progenitor populations: the c-kit^+^/Sca-1^+^/Lin^−^/IL-7R*α* ^+^ common lymphoid progenitor (CLP), which differentiate into B cells, T cells, NK cells, and a subset of dendritic cells (DCs), and the c-kit^+^/Sca-1^−^/Lin^−^/IL-7R*α* ^−^ common myeloid progenitor (CMP), which can populate the erythrocyte, megakaryocyte, myeloid-derived DC, granulocyte, and monocyte compartments [4, 5]. More specific precursors of the monocyte/macrophage lineage have been identified, including the c-kit^+^/Lin^−^/CX3CR1^+^ monocyte-macrophage DC progenitors (MDP) that give rise to both monocytes and dendritic cells [6]. Recent work has also identified a CD135^−^/Ly6C^+^ committed progenitor derived from the MDP that is restricted to the monocyte-macrophage lineage [7]. Mature CD11b^+^/CD115^+^ monocytes can then enter the circulation in order to be distributed around the body. Circulating monocytes are a heterogeneous population themselves, consisting of so-called patrolling monocytes and inflammatory monocytes [8]. Patrolling monocytes are responsible for crawling along the luminal side of the endothelium to monitor for danger-associated molecular patterns (DAMPs) and, upon encountering such a signal, rapidly entering the tissue and beginning to recruit additional effector cells in order to start a productive immune response [9]. A major function of inflammatory monocytes is to respond to sites of inflammation and tissue damage. Monocytes are recruited to these sites by following a variety of chemokine gradients, the most well-characterized of which is chemokine (C-C motif) ligand 2 (CCL2) [10, 11]. Upon arriving in the vasculature near the site of inflammation, monocytes begin a process of rolling adhesion in which selectin molecules on the surface of the endothelial cells bind to selectin ligands on the monocytes [12, 13]. These interactions then allow tight binding to occur between vascular cell adhesion molecule 1 (VCAM1) on the endothelium and integrin molecules on the monocytes [14–16]. Finally, the monocytes are arrested and can exit the circulation and enter the inflamed tissue, a process known as diapedesis [12]. Once in the tissue, monocytes can be further differentiated to macrophages in the presence of colony stimulating factor-1 (CSF-1) to carry out effector functions involved in pathogen clearance, wound healing, and developmental regulation [17, 18].

Macrophages are a cell type with exquisite plasticity and are able to carry out a diverse array of functions. In order to accomplish this, macrophages respond to signals from cytokines, chemokines, growth factors, and pathogen-derived factors in the microenvironment. In the early stages of an infection, macrophages are activated by interferons produced by infected cells and by bacterial-derived compounds such as flagella, lipopolysaccharide (LPS), and unmethylated CpG motifs [19–21]. These signals typically induce a proinflammatory response in macrophages to limit pathogen spread and recruit additional innate and adaptive immune cells to the site of infection. After the infection has been controlled and the pathogen cleared, macrophages are instrumental in the resolution of inflammation to prevent further tissue damage. Cytokines such as interleukin-4 (IL-4) and IL-13 can promote an anti-inflammatory response in macrophages to block additional activation of immune cells in the tissue and promote tissue remodeling and collagen deposition [22, 23].

## 2. Resident Macrophages in the Mammary Gland

### 2.1. Mammary Gland Development

In addition to their roles in pathogen clearance and wound healing, macrophages can also respond to cytokines present in the tissue microenvironment during development, where complex and reciprocal interactions take place between epithelial and stromal cells. One particular site where such interactions take place is in the developing mammary gland. Beginning early in embryogenesis, patterning of the mammary glands occurs with the specification of the sites of the developing glands [24–26]. As development continues, epithelial cells invaginate into the surrounding mesenchyme and form the mammary bud. Just prior to birth, the cells begin to proliferate and allow the bud to invade into the adjacent fat pad. Once this has occurred, the mammary epithelial cells (MECs) begin a process of ductal morphogenesis to generate a rudimentary ductal tree [27, 28]. A prominent structure in pubertal mammary gland development is the terminal end bud (TEB), the site of actively proliferating epithelial cells. These organized structures are found at the distal end of the mammary ducts and contain cap cells and body cells, which give rise to cells of the myoepithelial and luminal lineages, respectively [29, 30]. As the cells proliferate, the TEBs advance through the fat pad until they reach the edge, at which time they regress to form the terminal ducts. At this point, side branching occurs to create secondary and tertiary ducts from the main ducts to fill the entire fat pad laterally. The mammary gland undergoes large-scale expansions and regressions during repeated estrous cycles, with new epithelial buds sprouting from the ducts and subsequently disappearing as estrogen and progesterone levels rise and fall [31, 32]. During pregnancy, however, these hormone-induced changes stop being cyclical and the gland enters a state of preparation for lactation. Alveolar buds form in response to prolactin and develop into mature alveoli to produce milk [33, 34]. After weaning, the mammary gland must return to its resting, prepregnancy state through a tightly regulated process of programmed cell death called involution [35]. At this time, the mammary gland begins to expand and regress again during estrous cycles and is ready to expand again in response to another pregnancy.

During postnatal development, numerous cytokines and hormones regulate further growth of the mammary gland. Previous work has shown that cytokines IL-4 and IL-13 are critical for promoting the differentiation and maturation of luminal epithelial cells [36]. Additionally, the requirement of estrogen receptor (ER) and progesterone receptor (PR) signaling in pubertal development has been demonstrated through elegant tissue recombination studies. While embryonic development is unaffected, mammary glands of ER*α*-null mice fail to elongate through the fat pad during pubertal development and lack defined TEBs [37]. Despite this lack of outgrowth, ER*α*-null epithelium is still responsive to progesterone and form alveoli during pregnancy. The requirement of ER signaling is limited to the epithelial cells, as transplantation of wild-type MECs into an ER*α*-null fat pad results in normal ductal morphogenesis [37]. Additional studies have shown a differing role for PR signaling, with transplantation of PR-null MECs into wild-type fat pad resulting in the formation of a normal ductal tree [38]. As expected, however, PR-null MECs fail to respond to progesterone during pregnancy and do not form alveolar structures. Intriguingly, transplantation of wild-type MECs into a PR-null fat results in a modest defect in ductal outgrowth, suggesting a role for PR signaling in stromal cells regulating MEC proliferation in a paracrine manner [38]. Notably, ER and PR signaling promote MEC proliferation in a paracrine manner, with previous reports demonstrating that proliferating cells are not contained within the ER^+^ or PR^+^ compartments [39–41]. Hormone signaling is a tightly regulated process, with any deviations above or below the optimal levels resulting in similar defects. Exposure to exogenous estrogen treatment results in decreased ductal elongation, similar to results seen in ER*α*-null MEC transplants; however, estrogen treatment also leads to increased lateral branching [42]. Thus, keeping hormone levels and signaling within a specified range is of critical importance for maintaining mammary gland integrity.

### 2.2. Macrophages in the Developing Mammary Gland

As a cell type that serves to act as a first line of defense against foreign substances and pathogens, it is only logical to have macrophages dispersed throughout the body. But in addition to their role as immunological surveyors, macrophages also play critical roles in regulating mammary gland development. Previous studies have indicated that macrophages are found in close association with MECs at many well-characterized stages of mammary gland development [43]. Immunostaining of mammary glands for the macrophage marker F4/80 indicates the presence of macrophages surrounding the body cells of the TEB [43, 44]. These macrophages are poised to phagocytose cellular debris from MECs undergoing apoptosis while generating the hollow lumen of the mammary ducts [45]. At maturity, macrophages can be found lining the mammary ducts where they promote epithelial cell proliferation and differentiation through production of growth factors, chemokines, and inflammatory mediators. During lactation, F4/80^+^ macrophages have been observed in close proximity to the alveoli and are a major cellular component of milk [43, 44, 46]. Once lactation is completed and weaning occurs, the mammary gland undergoes involution to return to its prepregnant state, involving large amounts of apoptosis and extracellular matrix (ECM) remodeling. Again, macrophages are major contributors to this process, phagocytosing apoptotic cellular debris and producing matrix remodeling factors to facilitate the transition back to the fully involuted state [47, 48].

Numerous studies have been undertaken using genetic and biochemical approaches to deplete macrophages during mammary gland development. Mice homozygous for a null mutation in CSF-1, the critical factor required for macrophage differentiation, show significant impairment in ductal elongation during mammary gland development [49]. This defect can be rescued through the use of a tetracycline-inducible transgene to reexpress CSF-1. Architecturally, organization of collagen I into long fibers around the neck of the TEBs is impaired in CSF-1-deficient mice while total collagen I deposition is unaffected, implicating a specific role for macrophages in regulating collagen organization but not collagen biosynthesis [50]. The contributions of macrophages to estrous-cycle induced changes were described elegantly using the CD11b-DTR inducible mouse model of macrophage depletion. Macrophages are found at different frequencies in the mammary gland during the estrous cycle, reaching a maximum during diestrus. Depletion of macrophages resulted in a nearly 50% reduction in alveolar bud formation in response to progesterone treatment and an overall decrease in MEC proliferation [51]. Additional work using sublethal irradiation has demonstrated that cells of the hematopoietic lineage are required for the formation of TEBs during pubertal development and that macrophages modulate their immunostimulatory profile over the course of the estrous cycle [45, 52].

While these studies clearly demonstrate that a role for macrophages is regulating mammary gland development, the mechanism by which this occurs remains unclear. One possible mechanism is that macrophages in the microenvironment respond to the same cytokines and growth factors required for epithelial cell development and respond in a unique way. IL-4 and IL-13 have been implicated in mammary epithelial cell differentiation and are found at measureable amounts in the developing mammary gland [36]. When exposed to these cytokines, macrophages respond by producing a host of anti-inflammatory factors and tissue remodeling agents known to be needed during mammary gland development. Studies of macrophages in infection models have illustrated that tissue-resident macrophages are more predisposed to an anti-inflammatory response compared to monocyte-derived macrophages recruited from the circulation [53, 54]. Transforming growth factor-beta (TGF-*β*) and members of the matrix metalloproteinases (MMP) family are produced by macrophages at high levels in response to IL-4/IL-13 stimulation* in vitro *[19, 55]. In the setting of the mammary gland* in vivo*, MMPs are required to degrade and remodel the ECM to allow further ductal elongation to occur through the fat pad, while TGF-*β* plays a suppressive role to limit the extent of ductal branching [56–59]. Thus, it is possible that IL-4 and IL-13 play dual roles in the microenvironment: promoting MEC differentiation and stimulating tissue-resident macrophage function. While ductal elongation is driven primarily by ovarian-produced estrogen, studies in breast cancer have shown that macrophages themselves are capable of producing estrogen locally through the expression of the estrogen synthesizing enzyme aromatase [60]. There is a relative lack of knowledge to date regarding the role of macrophage-produced estrogen, but it is tempting to speculate that macrophages associated with the TEBs or lining the mammary ducts could regulate development and proliferation directly by creating pools of locally concentrated estrogen. Further studies are warranted to determine if macrophages express aromatase* in vivo* and how the resulting rise in estrogen levels in the mammary gland affects development. In addition, the increased estrogen and proliferative signals in the mammary gland may also help establish a protumorigenic environment, in which the MECs are primed for the tumor initiation when exposed to an oncogenic insult. Understanding how changes that take place in the mammary gland during development can affect tumor initiation at a later point in life is critical in developing preventative strategies through life-style changes and therapeutic intervention.

### 2.3. Effects of Inflammation on Resident Macrophages

**Figure 1:**
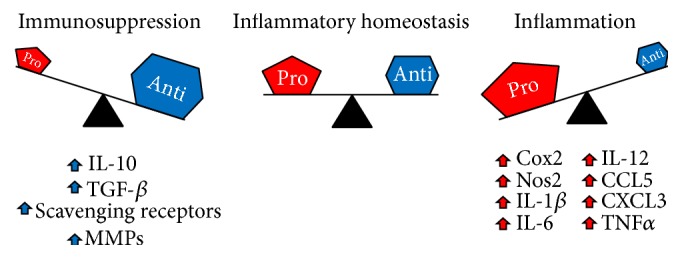
Tissue-resident macrophages are important for maintaining a state of inflammatory homeostasis. Under normal conditions, pro- and anti-inflammatory signals are maintained in a balanced state referred to as inflammatory homeostasis (center). During the early stages of infection or tissue damage, increased production of proinflammatory factors can tip the balance towards an overall inflammatory state (right). During late stages of infection and wound healing, the production of anti-inflammatory factors is significantly increased, leading to an immunosuppressive state (left). A failure to return to inflammatory homeostasis leads to chronic inflammation or immunosuppression and can lead to the development of numerous pathologies, including cancer.

Recent evidence has supported the long-postulated idea that chronic inflammation enhances the risk of developing cancer [61–64]. Furthermore, diseases with systemic inflammatory components are major risk factors for certain types of cancer, including breast cancer [61, 65]. In patients with Crohn's disease, increased expression of the proinflammatory cytokine tumor necrosis factor-alpha (TNF*α*) recruits inflammatory macrophages and leads to the production of additional proinflammatory factors, initiating a feed-forward loop which leads to tissue damage and predisposition to oncogenic initiation [66]. One of the most common diseases associated with cancer risk is obesity, with 34.9% of adults in the United States being classified as obese [67]. Patients with obesity often have elevated serum levels of proinflammatory molecules, such as IL-6, which induce a systemic chronic inflammatory state [68]. In the mammary gland microenvironment specifically, obesity is directly linked with increased IL-6 signaling and increased macrophage recruitment compared to normal-weight mammoplasty specimens [69]. In a resting state, the amount of proinflammatory and anti-inflammatory signals are maintained in a state of equilibrium (Figure 1). However, in pathologic settings such as obesity, inflammatory homeostasis is lost and the balance is tipped in favor of proinflammatory factors. In these cases, the increased abundance of proinflammatory factors relative to anti-inflammatory factors affects cells in the microenvironment. Once macrophages are exposed to proinflammatory factors they upregulate the production of additional proinflammatory factors, creating a feed-forward loop that further upsets inflammatory homeostasis. It is interesting to speculate why obese patients with increased levels of IL-6 have a predisposition to developing ER^+^ breast cancers specifically [70]. Studies focused on endometrial carcinoma have revealed a paracrine signaling axis whereby cancer cells produce IL-6 to stimulate stromal cells to upregulate aromatase and produce estrogen, thus inducing a cycle of increased cancer cell proliferation and IL-6 production [71]. It remains to be seen if a similar axis exists in breast cancer, but with their role in regulating mammary gland development, it is not difficult to hypothesize that macrophages may upregulate aromatase expression in response to IL-6 in the context of obesity, thus providing a mechanistic explanation of the propensity for obese women to develop ER^+^ breast tumors.

In addition to pathologic inflammatory conditions, acute inflammatory responses in the context of normal tissue processes can have profound impacts on the microenvironment. In the 5-year period following childbirth women are susceptible to developing postpartum breast cancer with a particularly poor prognosis [72]. Elegant xenograft studies in mice have revealed that the microenvironment of the involuting mammary gland significantly enhances tumor growth compared to nulliparous mammary glands [73]. Most recently, an overall profile was created to determine the relative abundance of immune cells during the process of involution compared to nulliparous and lactating glands. While modest changes were observed in DC recruitment at all time points of involution, a near 10-fold increase in macrophage recruitment is observed during the first week of involution and remains elevated at 4 weeks after weaning [47]. This increased macrophage recruitment was accompanied by increased CD4^+^ and CD8^+^ T cell recruitment and an increased presence of FOXP3^+^ regulatory T cells. Mechanistically, the microenvironment of the involuting mammary gland induces macrophages to take on an immunosuppressive profile by producing IL-10 and suppressing T cell activation [47]. This acute disruption of inflammatory homeostasis results in the formation of a protumorigenic niche through direct suppression of adaptive immunity. A better understanding of the critical balance between proinflammatory and anti-inflammatory factors is clearly needed in order to develop new therapeutic regimens for the treatment and even prevention of breast cancer.

## 3. Macrophages in the Tumor Microenvironment

In addition to their contributions to normal mammary gland development, macrophages are well-established constituents of the breast tumor microenvironment. Increased macrophage density in pretreatment biopsies of breast cancer patients correlates with reduced recurrence-free and overall survival [74–76]. Therefore, efforts have focused on understanding the mechanisms through which macrophages contribute to breast cancer growth and progression and these topics have been reviewed extensively [19, 55, 77–79]. Myeloid cells in the tumor microenvironment, in particular macrophages, have been shown to contribute to tumor growth and progression in a variety of ways. Tumor-associated macrophages (TAMs) secrete soluble factors, such as vascular endothelial growth factor (VEGF), which induce angiogenesis and partially relieve the hypoxic stress within fast-growing tumors [80]. In addition to promoting angiogenesis, TAMs support tumor cell survival, migration, and invasion through the secretion of growth factors such as EGF and FGFs and chemokines such as CXCL1/2 [81–84]. Of note, TAMs not only secrete factors but also facilitate the release of protumorigenic factors from the ECM, a topic discussed later in this review. In recent studies using intravital imaging techniques, Lohela et al. demonstrated that prolonged depletion of myeloid-derived cells in a model of breast cancer resulted in delayed tumor growth, decreased angiogenesis, and fewer lung metastases [85]. Furthermore, production of growth factors and ECM remodeling by TAMs have been implicated in promoting breast cancer resistance to chemotherapeutic agents such as paclitaxel, doxorubicin, and etoposide (reviewed in [86]). Finally, numerous studies have provided evidence of TAMs interacting with cells of the adaptive immune system, mainly CD4^+^ and CD8^+^ T lymphocytes, and both directly and indirectly suppressing their antitumor effects [87–89].

Understanding macrophage functions in the context of normal tissue development can provide insights into the functions of macrophages during tumor growth and progression. Specifically, there are parallels between the mechanisms of macrophage recruitment and macrophage-mediated alterations in ECM in both the normal mammary gland and the tumor microenvironment. Furthermore, it is becoming clear that the balance between proinflammatory and anti-inflammatory factors is key to the regulation of macrophage function within the tumor microenvironment. Therefore, further discussion will focus on macrophage recruitment, polarization, and regulation of ECM within the tumor microenvironment.

### 3.1. Recruitment of Macrophages to the Tumor Microenvironment

As mentioned above, CCL2 and CSF-1 are important for both recruitment and differentiation of macrophages in the normal mammary gland. Likewise, these factors have been implicated in recruitment of macrophages to both primary and metastatic tumor sites. Using genetic approaches, seminal studies demonstrated that CSF-1 is critical for macrophage recruitment and differentiation in tumor microenvironment of MMTV-PyMT mice [90]. These studies demonstrated that reduced macrophage infiltration significantly reduced the ability of the tumor cells to metastasize to the lung. Tumor cell-derived CSF-1 has also been linked to the proliferation of a protumor subset of CD11b^lo^F4/80^hi^ macrophages in the MMTV-Neu transgenic model of mammary tumor growth [91]. In these studies, administration of the CSF-1R inhibitor GW2580 to tumor bearing mice drastically reduced the numbers of CD11b^lo^F4/80^hi^ macrophages in S phase. These, and other recent studies, suggest that in addition to recruitment of monocytes from the bloodstream, certain TAM populations are able to proliferate within the tumor microenvironment [91–93]. Taken together, these studies indicate that therapies aimed at targeting the accumulation and/or proliferation of TAMs may improve clinical outcomes for breast cancer patients, and as a result CSF-1R inhibitors and blocking antibodies have entered clinical trials for various cancer types, including breast cancer. In a recent report, Ries et al. described a significant depletion of CD68^+^/CD168^+^ macrophages in a small cohort of breast cancer patients and among those receiving the highest protocol dose, analysis revealed a switch of lymphocyte infiltrates from CD4^+^ T cells before treatment to CD8^+^ T cells after treatment [94]. This study provides proof-of-principal that blockade of the CSF-1/CSF-1R pathway results in fewer macrophages recruited to human breast tumors, and this change in myeloid recruitment affects the overall composition of the tumor microenvironment.

Another key chemokine that has been implicated in macrophage recruitment to the tumor microenvironment is CCL2/MCP-1. Numerous studies have found that tumor cell-derived CCL2 promotes macrophage recruitment both* in vitro* and* in vivo* [95–97]. In recent studies, both CCL2 and CCL5/RANTES were found to correlate with increased macrophage recruitment in human patient samples, and specifically in ER^+^ samples [98]. Using estrogen-supplemented oophorectomized mice bearing MMTV-PyMT mammary tumors, further studies demonstrated that inhibition of either CCL2 or CCL5 using blocking antibodies resulted in reduced macrophage infiltration and reduced tumor growth [98]. In addition to promoting recruitment of macrophages to the primary tumor site, CCL2 has also been implicated in indirectly promoting the seeding and growth of tumor cells in the metastatic site. Specifically, CCL2 was found to recruit a distinct population of macrophages termed metastasis-associated macrophages, defined as CD11b^+^Ly6C^high^, to the lung metastatic site [10]. Once localized to this site, CCR2 activation stimulates macrophages to secrete an additional chemokine, CCL3, which contributes to tumor cell-macrophage interactions and retention in the metastatic site through activation of CCR1 [99]. Taken together, these studies suggest that blocking macrophage recruitment through inhibition of chemokine signaling may effectively reduce macrophage contributions during tumor growth and progression. However, some challenges have been associated with targeting chemokines including the induction of compensatory mechanisms in response to chemokine inhibition. In a recent study evaluating CCL2 blockade, Bonapace et al. found that while blocking CCL2 reduced lung metastasis, which was maintained upon continuous CCL2 inhibition, cessation of CCL2 neutralization led to increased metastasis and accelerated death [100]. Assessment of combinatorial therapies, which included targeting additional cytokines, such as IL-6, that were increased in the lungs upon treatment cessation, alleviated the increase in metastasis. Thus, these studies suggest that targeting chemokines, such as CCL2, as a therapeutic strategy should be approached with caution and could possibly require combination-based approaches for success.

In addition to CSF-1 and CCL2, other chemokines have also been linked to macrophage recruitment in the primary tumor site. Using an inducible model of mammary tumorigenesis, we identified CX3CL1 as a mediator of macrophage recruitment to early stage mammary hyperplasias [101]. More recent studies have linked CX3CL1 expression with poor outcome in breast cancer patients [102], although whether high CX3CL1 is linked to macrophage recruitment in human breast cancer samples remains to be determined. Boyle et al. recently reported that CCL20-CCR6 axis is important for regulating macrophage recruitment into mammary tumors of MMTV-PyMT mice [103]. In these studies, growth of mammary tumors in CCR6-knockout mice led to reduced mammary tumor initiation and growth. Further analysis of these tumors revealed a reduction in immune cell infiltration along with changes in macrophage polarization as shown by reduced expression of IL-4R and CD206. Importantly, reconstitution of TAMs into CCR6-knockout mice bearing orthotopically transplanted MMTV-PyMT tumors restored tumor growth demonstrating the importance of this chemokine axis for mammary tumor growth. In addition to general recruitment to the tumor microenvironment, a subpopulation of macrophages is also known to accumulate in hypoxic regions within tumors. Recruitment of macrophages into hypoxic regions is mediated through soluble factors such as VEGF, endothelin-2, and angiopoietin-2 [104, 105]. Semaphorins, such as Sema3A, were recently linked to recruitment of macrophages to hypoxic regions via a neuropilin-1-dependent mechanism [106]. Additional recent studies have also found that hypoxic cancer cells produce chemoattractants that promote macrophage recruitment, including oncostatin M and eotaxin, which also act to polarize macrophages to a protumor phenotype and are required for tumor progression [107]. Taken together, these studies demonstrate that macrophage recruitment into the tumor microenvironment can be driven by many different factors, highlighting the complexity of the mechanisms driving macrophage infiltration.

Although less extensively studied compared with tumor cell-derived chemokines, stromal cells, including carcinoma associated fibroblasts (CAFs), mesenchymal stem cells (MSCs), and endothelial cells, also produce chemokines that can potentially recruit macrophages into the microenvironment. Stimulation of CAFs and MSCs with tumor cell-derived conditioned media leads to upregulation of various chemokines, including CCL2, CXCL8, and CCL5 [108]. Furthermore, Yoshimura et al. demonstrated that stromal cell-derived CCL2 contributes to macrophage recruitment to 4T1 tumors and that loss of stromal cell CCL2 leads to decreased lung metastasis [109]. Recent genetic studies have demonstrated a critical role for BMP signaling in the regulation of chemokines from fibroblasts. Specifically, loss of BMPR2 from fibroblasts led to increased metastasis of MMTV-PyMT tumors corresponding with increased chemokine expression and increased infiltration of myeloid cells [110].

**Figure 2:**
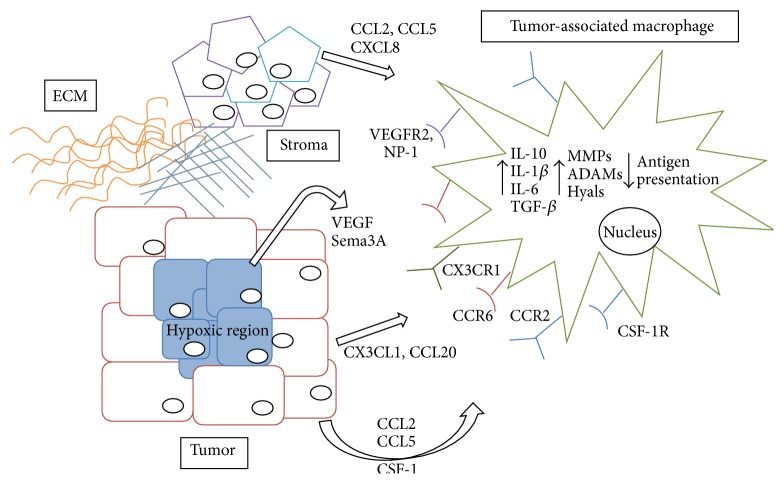
Complex interactions in the tumor microenvironment. Breast cancer cells located in the tumor periphery (red rectangles) secrete cytokines and chemokines, which recruit monocytes from the circulation and differentiate them into tumor-associated macrophages (TAMs). Tumor cells located in the inner, hypoxic region (blue rectangles) develop a more specialized array of molecules to recruit macrophages poised to help the hypoxic cells survive and proliferate. The stromal cells of the tumor, along with components of the extracellular matrix (such as collagen I and hyaluronan), additionally contribute to the recruitment and retention of TAMs. Once educated by the tumor microenvironment, TAMs upregulate pathways associated with both M1- and M2-activated macrophages and actively support the survival, proliferation, and metastasis of breast cancer cells.

In addition to chemoattractants derived from tumor and stromal cells, there is evidence that tumor-associated ECM may also contribute to macrophage recruitment. For example, collagen fragments are known to be chemotactic for inflammatory cells [111]. Furthermore, it has been proposed that proteolysis of collagen I promotes macrophage recruitment into the involuting mammary gland, which is characterized as a tumor-promoting environment [112]. Another ECM component linked to macrophage recruitment is hyaluronan, which is a glycosaminoglycan consisting of repeating disaccharide subunits of glucuronic acid and N-acetylglucosamine. Macrophages are often associated with a hyaluronan-containing matrix within the tumor environment, and studies have suggested that hyaluronan can act directly on macrophages to regulate their migration [113]. Specifically, hyaluronan has been shown to promote macrophage chemotaxis using* in vitro* chemotaxis assays [114]. Consistent with these findings,* in vivo* studies have demonstrated that reduction of hyaluronan in the mammary tumor stroma correlates with decreased macrophage infiltration [115]. Taken together, the numerous studies focusing on macrophage recruitment demonstrate that macrophage infiltration into the tumor microenvironment can potentially be mediated by a variety of factors (Figure 2). Further studies are warranted to understand the relative contributions of tumor cell versus stromal cell derived chemokines and ECM components to macrophage recruitment during tumor growth and progression.

### 3.2. Macrophage Polarization within the Tumor Microenvironment

Once recruited to the tumor microenvironment, macrophages respond to the plethora of stimuli within the microenvironment and differentiate into various effector subsets. Numerous studies have focused on defining macrophage subsets within the tumor microenvironment. Currently, the most widely accepted classification of macrophage polarization is based on descriptions of classical (M1) versus alternative (M2) polarization, which were developed as a result of initial studies investigating macrophage responses to helper T cells 1 (Th1) and helper T cell 2 (Th2) derived molecules [116]. Classically activated macrophages develop in response to interferon-gamma (IFN*γ*) and pathogen-derived toll-like receptor ligands [19, 117]. This response is characterized by the production of cytotoxic factors such as reactive oxygen species and nitric oxide, increased rates of phagocytosis, and enhanced antigen presentation on the cell surface. Alternatively activated macrophages, on the other hand, develop as part of the wound healing program and as such are thought to antagonize inflammation. M2 macrophages are induced by the Th2 cytokines IL-4 and IL-13, as well as in response to IL-10, immunoglobulins, and glucocorticoids [55, 118]. These cells, in turn, secrete factors that promote angiogenesis, upregulate expression of scavenging receptors, and produce enzymes to remodel the surrounding extracellular matrix. As interest and work in the field of macrophage biology has expanded, the nomenclature describing the activation status of macrophages has become complex and often confusing. In an attempt to streamline the methods used to generate and describe the cells used by the different research groups, Murray et al. published a comprehensive set of recommendations which will undoubtedly simplify future analysis and comparison of macrophage subsets [119].

Based on their functions within the tumor microenvironment, TAMs have been generally characterized as M2-like [55]. Several studies have demonstrated that TAMs express higher levels of scavenging receptors, angiogenic factors, and proteases, similar to M2 macrophages. Furthermore, TAM polarization to the M2-like phenotype in the MMTV-PyMT model has been attributed to IL-4-producing Th2 cells within the tumor microenvironment [89]. However, there is evidence that macrophages exhibit different phenotypes during different stages of tumor initiation and progression. During early stages of transformation, recently recruited macrophages are exposed to a wide variety of proinflammatory signals derived from the epithelial cells and the surrounding stroma and often express M1-related factors that have protumorigenic properties, such as IL-1*β* and IL-6 [120, 121]. As a component of the proinflammatory response, production of reactive oxygen and nitrogen species could also potentially enhance the rate of epithelial cell mutation and thus accelerate tumorigenesis [122]. In established tumors, macrophages exhibit alternatively activated functions including the production of immunosuppressive factors, such as IL-10 and TGF-*β*, which are capable of actively suppressing the antitumor immune response [79, 88, 89]. These macrophages also produce growth factors and remodel the matrix, supporting tumor cell growth and enhancing invasion. Therefore, TAM phenotypes are now thought to include a combination of markers typically assigned to the M1 and M2 phenotypes. Thus, as efforts are being made to “repolarize” macrophages within the tumor microenvironment towards the M1/classically activated phenotype, care must be taken to ensure that the potentially protumorigenic functions of these macrophages are suppressed.

Recent sophisticated analyses utilizing genomewide studies and RNA-sequencing have revealed that macrophage phenotypes* in vivo *are far more heterogeneous and complex than initially expected. Xue et al. performed a detailed transcriptome analysis of primary human monocytes stimulated with 28 different signals, the results of which suggest a “spectrum” model where 9 different macrophage activation programs were identified in response to different combinations of stimuli [123]. Analysis of the enriched gene sets in human macrophages derived from smokers and COPD patients revealed activation programs within these primary macrophages that were significantly different from the hypothesized phenotypes. In smokers' samples, a complex network of stimuli including glucocorticoids, free fatty acids, and IL-4 were detected, while in COPD patient samples the previously published IL-4/IL-13 associated gene signatures were not reproduced and instead a profound loss of inflammatory genes was reported [123]. These results demonstrate the complexity of activating signals responsible for the phenotypes of macrophages in human pathologies, and they suggest that a simple bipolar M1/M2 paradigm may not be sufficient to describe macrophages associated with disease states. Based on the observation that the microenvironment of lung disease is capable of producing a spectrum of macrophage activation states, it seems likely that this heterogeneity would also be observed in the tumor microenvironment. Indeed, while performing gene-expression profiling on TAMs and mammary tissue macrophages from tumor bearing MMTV-PyMT mice, Franklin et al. observed few canonical M2 markers to be upregulated in the TAM population [92]. Instead, they reported TAM differentiation to be dependent on signaling of the transcription factor* Rbpj*, a key regulator of canonical Notch signaling.

In addition, recent evidence suggests that individual tumors may contain several different subsets of macrophages and those might differ in their functions. Movahedi et al. reported the presence of two distinct TAM populations in mammary TS/A tumors, distinguishable most easily by the level of MHCII expression on their surface [124]. MHCII^lo^ macrophages were shown to reside mainly in hypoxic tumor regions and expressed markers associated with M2 polarization. The MHCII^hi^ subset, however, expressed M1-signature genes such as* Cox2, Nos2,* and* IL-12*. These cells were shown to secrete proinflammatory cytokines and chemokines such as IL-6, CCL5, and CXCL3, which could in turn serve to further recruit additional proinflammatory cells to the tumor margins. However, both macrophage subsets were shown to be poor antigen presenting cells and were able to suppress T cell proliferation, indicating that both subsets might be capable of contributing to protumor immunosuppression. Interestingly, Ruffell et al. observed a similar localization of MHCII^lo^ and MHCII^hi^ TAMs in mammary tumors derived from MMTV-PyMT mice; however, the ability of TAMs to suppress CD8^+^ T cell proliferation was limited to the MHCII^lo^ subset of cells [88]. These findings indicate that some TAM properties are most likely universal (recruitment, localization), whereas other properties (specific interactions with other infiltrating cells) might be dependent on the tumor model under investigation. In a recent study examining macrophage localization within human breast tumors, high CD68^+^ macrophage staining within gaps of ductal tumor structures correlated with reduced lymph node metastasis [125]. Taken together, these data suggest that TAMs represent a macrophage population that is distinct from both M1 and M2 macrophages as they are canonically described in the setting of infection, but there is most likely a spectrum of TAMs whose phenotype and function depend on tumor type and location within the tumor.

### 3.3. Macrophage Regulation of ECM within the Tumor Microenvironment

One of the identified mechanisms through which macrophages may regulate ductal elongation during mammary gland development is through organization of ECM, such as collagen [50]. While some functions of TAMs in the tumor microenvironment, including promotion of tumor cell migration and invasion, angiogenesis, and suppression of adaptive immune responses, have been extensively examined, the contributions of macrophages to the modulation of ECM remain relatively understudied. Macrophages actively contribute to the changes in ECM through the production of ECM components and through the release of factors that cleave ECM. Consequently, ECM components and their fragments can act directly on macrophages to promote their recruitment, retention, and function. One of the mechanisms through which alternatively activated macrophages contribute to resolution of inflammation is through producing and remodeling the ECM. Therefore, it is not surprising that alternatively activated macrophages produce ECM. Macrophages have been found to produce fibronectin [126] and collagen [127], including high levels of type VI collagen, which is increased in alternatively activated macrophages and promotes monocyte adhesion [128]. While studies focusing on the contributions of macrophages to ECM deposition in the context of breast cancer are limited, it is worth noting that collagen VI is found at the invasive edge of breast tumors, where macrophages are known to localize, and promotes epithelial-mesenchymal transition of cancer cells [128]. Thus, studies aimed at determining whether macrophages at the leading edge contribute to these high levels of collagen VI are warranted.

In addition to producing ECM, macrophages express high levels of proteases that can contribute to the cleavage and remodeling of ECM. Gene profiling of TAMs demonstrates increased expression of proteases including MMPs, ADAMs, and cathepsins [81]. Macrophage-derived proteases can contribute to protumor alterations in the stroma in a number of ways including facilitating ECM breakdown for subsequent invasion and migration, liberation of tumor-promoting factors from the ECM, and generation of bioactive ECM fragments. For example, macrophage-derived MMPs have been linked to the release of angiogenic factors, such as VEGF and FGFs, in the tumor microenvironment [81]. In addition, studies demonstrated that alternatively activated macrophages are directly involved in collagen turnover, specifically through uptake and degradation of collagen by CX3CR1-positive cells involving the mannose receptor [129]. Uptake of collagen requires MMP activity, potentially linking macrophage regulation of collagen to both cleavage and uptake.

Macrophages may also regulate hyaluronan in the tumor microenvironment. Hyaluronan is generated as a high molecular weight glycosaminoglycan that can be broken down into fragments that are characterized as inflammatory and protumorigenic [113]. Hyaluronan cleavage occurs through enzymatic degradation by hyaluronidases (Hyals) or by mechanisms involving reactive oxygen and nitrogen species [130]. Macrophages have been found to express hyaluronidases [131] and can thus potentially contribute to the breakdown of hyaluronan during inflammation and tumor progression. In turn, it has been suggested that hyaluronan can direct macrophage function. Exposure of macrophages to hyaluronan, either purified or tumor-derived, leads to increased expression of various inflammatory mediators including IL-1*β* [132] and IL-10 [133]. In recent studies, tumor-derived microvesicles were found to induce IL-10 expression in macrophages using a hyaluronan-dependent mechanism through the PI3K/Akt/mTOR pathway [134]. Together, these studies suggest a link between hyaluronan and regulation of macrophage function, possibly through enhancing immunosuppressive function. Together, these observations suggest that macrophages are likely to be important regulators of ECM production and remodeling in the tumor microenvironment, and further studies are warranted to define the specific functional consequences of these actions.

## 4. Summary

In conclusion, it is clear that inflammation is a complex process that has evolved to resolve damage to the body caused by pathogens or disease. In the normal mammary gland, tissue-resident macrophages play a vital role in the regulation of development and maintenance of tissue homeostasis. Pro- and anti-inflammatory factors produced in the microenvironment act not only on epithelial cells, but also on macrophages and lead to the further disruption of inflammatory homeostasis and the creation of a protumorigenic niche that is primed for oncogenic initiation. Tumor cells acquire the capacity to harness the functions of inflammatory cells, such as macrophages, to aid in their growth and progression. Experimental studies have demonstrated that macrophages interact with cancer cells and their phenotype and function evolve as the tumor itself evolves. However, recent studies demonstrating the complexity of macrophage polarization and the impact of macrophage localization within the tumor microenvironment suggest that the contributions of macrophages to breast cancer growth and progression are likely to be quite complex. Therefore, it will be critical to obtain a better understanding of the mechanisms that drive macrophage recruitment, polarization, and function within the tumor microenvironment at different stages of breast cancer formation and progression.
